# Oral Administration of *Bacillus toyonensis* Strain SAU-20 Improves Insulin Resistance and Ameliorates Hepatic Steatosis in Type 2 Diabetic Mice

**DOI:** 10.3389/fimmu.2022.837237

**Published:** 2022-02-15

**Authors:** Zhihua Ren, Samuel Kumi Okyere, Lei Xie, Juan Wen, Jiayi Wang, Zhengli Chen, Xueqin Ni, Junliang Deng, Yanchun Hu

**Affiliations:** ^1^Key Laboratory of Animal Disease and Human Health of Sichuan Province, Sichuan Agricultural University, Chengdu, China; ^2^Key Laboratory of Animal Diseases and Environmental Hazards of Sichuan Province, College of Veterinary Medicine, Sichuan Agricultural University, Chengdu, China; ^3^New Ruipeng Pet Healthcare Group Co., Ltd. Shenzhen, China

**Keywords:** type 2 diabetes, hepatic steatosis, *Bacillus toyonensis* SAU-20, lipogenic genes, insulin resistance

## Abstract

In this study, the ameliorative effects of *Bacillus toyonensis*-SAU-20 (*B. toyo* SAU-20), a new probiotic strain isolated and identified by our laboratory from *Ageratina adenophora*, on the development of insulin resistance and hepatic steatosis in type 2 diabetic (T2DM) mice was investigated. Thirty Specific-pathogen free Kunming (SPFKM) mice were randomly allocated to three groups: control, high fat diet/streptozotocin (HFD/STZ), and HFD/STZ+*B. toyo* SAU-20 groups with oral administration of *B. toyo* SAU-20 for 35 days. Biochemistry parameters, glucose tolerance, and insulin resistance were measured in the blood whereas histological analysis, inflammatory cytokines and lipogenic genes in the liver tissues. The results showed that, the levels of serum glucose, lipid profile, mRNA expression of lipogenic related genes and pro-inflammatory cytokines were significantly increased in T2DM mice. However, after *B. toyo* SAU-20 administration, the elevation of these parameters was significantly suppressed (P<0.05). In addition, the feeding of *B. toyo* SAU-20 significantly improved the morphological changes of the liver with significant alleviation of dyslipidemia, oxidative stress status and inflammation (P<0.05) indicating the ameliorating effect of *B. toyo* SAU-20 in hepatic steatosis in T2DM. Therefore, we concluded that, *B. toyo* SAU-20 alleviated insulin resistance and hepatic steatosis by improving the lipid profiles, antioxidant status and downregulating lipogenic genes as well as pro-inflammation cytokines expression.

## Introduction

Type 2 diabetes mellitus (T2DM) is a metabolic disorder that possess threat to human and animals (1). Reports have shown that by 2030 the world diabetes population would reach 439 million (2). In addition, developing countries are highly prone to this metabolic disorder in the near future according to the World Health Organization (3). People suffering from T2DM have higher risk (35-80%) of developing hepatic steatosis compared to diabetic free individuals (4). Insulin resistance is highly associated with metabolic syndrome, non-alcoholic fatty liver (NAFLD) (5). The liver is associated with the maintenance of lipid and energy homeostasis, and storage of excessive lipids in the liver as fat droplets, which when accumulated results in oxidative stress, inflammation, insulin resistance and diabetes (6). Furthermore, another key metabolic concern of diabetes is the unusual deposition of triglycerides in hepatocytes, which further stimulates hepatic steatosis (7).

*B. toyo* SAU-20 isolated from *Ageratina adenophora* an invasive plant (8–10) has showed good functional properties such as improving growth performance, antioxidant capacity, and gut integrity (11), of which are evident for the potential beneficial activity of *B. toyo* SAU-20 *in vivo*. This study was performed to examine the effect of *B. toyo* SAU-20 on fatty acid profile and liver functions in the HFD/STZ-induced T2DM in mice in order to reveal the treatment effects of probiotics in liver disorders such as NAFLD and its potential mechanisms, such as insulin resistance, hyperglycemia, oxidative stress, and inflammation.

## Material and Methods

### Sample Collection

Culture media were purchase from Qingdao Hope Bio-Technology Co., Ltd., Qingdao, China and Streptozotocin (STZ) was purchased from Solarbio solabao Beijing solabao Technology Co., Ltd, China. Mice, basal and high fat diet (**Table 1**) were purchased at the Chengdu Dashuo Experiment Animal Co. Ltd. Chengdu, China. *B. toyo* SAU-20 (Accessory No. MW287199) was obtained from the College of Veterinary Medicine (Professor Yanchun Hu’s lab), Sichuan Agricultural University, China.

**Table 1:** Feed composition.

Normal diet	Content g/kg	High fat diet	Content g/kg
Ingredients		Ingredients	
Water	94	Water	93
Carbohydrate	675	Carbohydrate	342
Protein	190	Protein	134
Fat	51	Fat	143
Fiber	36	Fiber	27
Ash	62	Ash	44
Calcium	11.3	Calcium	8.3
Phosphorus	8.6	Phosphorus	7.1

### Preparation of Probiotic Bacteria Suspensions

*B. toyo* SAU-20 were prepared by culturing in LB broth and incubating anaerobically at 37°C for 72 h. The bacterial cells were separated by centrifugation (3500g, 5 min) to remove the LB broth, and washed twice in 0.85% NaCl (Sigma), and then resuspended in 0.85% NaCl to a final concentration of 10^7^ CFU/mL and stored at 4°C.

### Experimental Animal and Design

Thirty (30) Specific-pathogen free Kunming (SPFKM) male mice (5 weeks old; BW 25-30 g) were purchased from the Chengdu Dashuo Experiment Animal Co. Ltd. Chengdu, China. The animals were kept in plastic cages in an animal house maintained with a constant temperature (22 ± 2°C) and humidity (65 ± 5%) under a 12-h light/12-h dark cycle. Mice were fed and given water *ad lib*. This study was approved by the Institutional Animal Care and Use Committee of Sichuan Agricultural University, Sichuan, China, under the permit number DKY-B2019603005.

The mice diabetes model was established by the feeding of high-fat diet (HFD) for 6 weeks and the intraperitoneal injection of streptozotocin (STZ) solution (dissolved in a 0.01 M citrate buffer, pH 4.5; Solarbio Science and Technology Co. Ltd., Beijing, China) with the dose of 35 mg/kg body weight for 3 consecutive days on the last day of the 6^th^ week (12). 72 h after the injection, fasting blood glucose (FBG) was measured using a blood glucose meter (Bayer). The diabetes model was recognized as successfully established when the blood glucose level was >11.1 mmoL/L (13). Mice injected with equal amounts of precooling citrate buffer solution, pH 4.5 were used as controls (n = 10). Immediately, the model mice were randomly divided into a Diabetic group (DBG) (n = 10) that was fed HFD + 1mL 0.9% normal saline daily oral gavage and Diabetic + *B. toyo* SAU-20 group (DBG + *B. toyo* SAU-20) (n = 10) that was fed HFD +1 mL of 10^7^ CFU/mL *B. toyo* SAU-20 oral gavage aside the control group (C) (n=10) fed basal diet and 1ml of 0.9% normal saline oral gavage for 5 weeks starting from the day of allocation of mice to their various groups. Feed and water intake was monitored and recorded daily throughout the experimental period. Feed and clean water was provided ad libitum. Water bottles were washed every week and fresh drinking water was placed in it for the next week’s administration. The bedding material (wood shavings) was also changed weekly. To administer the *B. toyo* SAU-20, new stocks were generated each week in LB and their viability was monitored by serial dilution and viable cell count using LB agar respectively.

### Oral Glucose Tolerance Test

At the last day of the last week of the administration of *B. toyo* SAU-20, oral glucose tolerance test (OGTT) was performed. Mice were fasted for 12 h and blood glucose values were determined (time = 0 min). Then mice were orally administered glucose (1 g/kg BW) and blood glucose levels were measured at 30, 60, 90 and 120 min from the tail vein using the glucose meter. Blood was collected from the tail vein using the syringe and few drops (2-5 µL) of the blood was immediately poured on the test strips of the glucose meter for the reading of glucose level in the blood. The sensitivity of the test strip was 0.1mmol/L.

### Blood and Tissue Sample Collection

At the end of the experiment (week 12), mice were fasted for 12 h and were anesthetized in an anesthesia chamber filled with 2.5% sevoflurane of O_2_ at a flow rate of 0.9−1 L/min for up to 6 hr (14). Blood samples were collected from the inferior vena cava and centrifuged at 4000 × g for 10min at 4°C, and the separated serum was stored at –80°C for further assays. Tissue samples (liver) were immediately removed, rinsed, and stored at –80°C or fixed in 10% paraformaldehyde solution.

### Biochemical Parameters

Glycogen and insulin were measured using ELISA kits (Jiangsu Jingmei Biological Technology Company Limited, Jiangsu, China). Lipid profiles, including total cholesterol (TC), total triglyceride (TG), LDL-cholesterol (LDL-C), and HDL cholesterol (HDL-C) were measured by commercial kits (Jiangsu Jingmei Biological Technology Company Limited, Jiangsu, China). Homeostatic model assessment of insulin resistance (HOMA-IR), used to quantify insulin resistance, was calculated as: HOMA-IR = Fasting blood glucose (mmol/L) × Fasting blood insulin (mU/L)/22.5 (15, 16). The levels of glycogen, glutathione (GSH), and malondialdehyde (MDA), and the activities of superoxide dismutase (SOD) in mice serum and livers were determined using commercial kits from Jiangsu Jingmei Biological Technology Company Limited, Jiangsu, China.

### Liver Histological Analysis

Livers were fixed into 4% parafomaldehyde. Then the tissues were embedded in paraffin and sectioned for 5 μm thick. Hematoxylinensin (H&E) staining was used for liver pathological evaluation. H&E staining kits were obtained from Jiancheng Bioengineering Institute (Nanjing, China). All kits were used according to the corresponding manufacturers’ instructions Liver steatosis score was numerically recorded following the method of Qayyum et al. (17).

### Enzyme-Linked Immunosorbent Assay

Liver tissues were washed with PBS, 0.1 g of the sample tissue was weighed and homogenized with 0.9 mL ice-cold PBS in a glass homogenizer, and then the mixture was centrifuged (3000 rpm, 20 min) to obtain the supernatant. Furthermore, we determined the protein concentration in the supernatant using a Total Protein Assay kit (Nanjing Jiancheng Bioengineering Institute, Nanjing, China). The supernatants were used to determine the concentrations of IL-1β, TNF-α, and IL-10 using a commercial mice ELISA kit (Jiangsu Jingmei Biological Technology Co. Ltd, Jiangsu, China), respectively. The level of sensitivity of each kit was 0.1pg/mL for each cytokine (18).

### Reverse Transcription-Quantitative Polymerase Chain Reaction (RT-qPCR)

Samples of liver tissues (30mg/mouse) were snap-frozen with liquid N_2_ and using a ceramic mortar, tissues were grinded into powder. Following the manufacturer’s instructions. total RNA from each sample was extracted using an Animal Total RNA Isolation Kit (Sagon Biotech, Shanghai, China). After validating the isolated RNA concentration and purity using the NanoDrop One system (Thermo Fisher Scientific, Waltham, MA; OD260/280 ≈ 1.9-2.0), cDNA was prepared form triplicate aliquots (each 1µg) using a PrimeScrip RT reagent kit (Takara, Tokyo Japan). Thereafter, qRT-PCR was performed using a SYBR Premix ExTaq (Takara) and a CFX96 thermal cycler (BioRad, Hercules, CA). The PCR conditions were shown as follows: 95°C for 5min, followed by 40 cycles of 95°C, 15 s for denaturation, 60°C, 60s for annealing at and 70°C, 25s for extension. Each qRT-PCR reaction was performed with volumes of 10 µL containing 5 µL TB Green TM Premix (Takara), 1 µL forward and reverse primers, 1 µL cDNA, and 2 µL DNase/RNase-Free Deionized Water (Tiangen, Beijing, China). The primers used to analyze the genes of interest were designed from NCBI genBank and are shown in **Table 2**. Relative gene expression in each sample was normalized to an internal control (β-actin); data analysis was performed using the 2−^ΔΔCt^ method. All samples were evaluated in triplicate.

**Table 2:** Primers used for the real-time PCR analysis.

Gene Name	Primer	Sequence (5’ and 3’)	Product length (bp)	Annealing Temperature (°C)	Sequence number
IL-1β	Forward	TGAAATGCCACCTTTGACAGTG	141	60.18	NM_008361.4
	Reverse	ATGTGCTGCTGCGAGATTTG			
PPAR-α	Forward	CTGCAGAGCAACCATCCAGA	109	60.04	XM_006520623.5
	Reverse	TGATGACCTGTACGAGCTGC			
IL-10	Forward	GGGGCGAGTGTAACAAGACC	109	60.27	XM_036162094.1
	Reverse	GCAGAGGAGGTCACACCATTT			
TNF-α	Forward	CCCTCACACTCACAAACCAC	211	59.82	NM_001278601.1
	Reverse	ATAGCAAATCGGCTGACGGT			
Acox1	Forward	AGGGAATTTGGCATCGCAGA	83	60.03	NM_001271898.1
	Reverse	AGGCCAACAGGTTCCACAAA			
Fgf21	Forward	CCGCAGTCCAGAAAGTCTCC	97	60.00	NM_020013.4
	Reverse	TGGCTGTTGGCAAAGAAACC			
FAS	Forward	TGGAATACACAGCCACCGAC	205	60.04	XM_030245556.1
	Reverse	AGGGCAGCTACCATGTTGTC			
SREBP-1	Forward	CGGCTCTGGAACAGACACTG	81	60.11	NM_001313979.1
	Reverse	TGAGCTGGAGCATGTCTTCG			
β-actin	Forward	TTCGCGGGCGACGAT	297	58.57	NM_0077393.5
	Reverse	CATCTTTTCACGGTTGGCCT			

### Statistical Analysis

Statistical analysis of the data collected (from various independent experiments) was performed using GraphPad Prism 5.04 software (GraphPad Software, Inc., La Jolla, CA, USA) and SPSS 20 Statistical Analysis Software (SPSS Inc., Chicago, IL, USA). The Shapiro-Wilk Test was used to test the normality of the data. All experimental results are presented as mean ± SD, and statistical significance were determined by one-way analysis of variance (ANOVA) followed by the Tukey’s test. The values were significantly different at P< 0.05.

## Results

### Effects of *B. toyo* SAU-20 on Growth Performance in HFD/STZ-Induced T2DM Mice

From the results, during the experimental trail we observed a significant increase in feed and water intake in the DBG group compared to the control (C) and *B. toyo* SAU-20 groups (**Table 3**, P<0.05), typical of T2DM. However, the level of feed and water intake in the *B. toyo* SAU-20 was higher compared to the control group. (P<0.05). Furthermore, we observed a decrease in the weight gain in the DBG group after the 35-days compared to the control (C) and *B. toyo* SAU-20 groups even though feed intake was high in the DBG group (P<0.05). No difference existed in weight gain between the control (C) and *B. toyo* SAU-20 groups. Moreover, we observed a significant decrease in liver, kidney and spleen indices in the diabetes group compared to the control and *B. toyo* SAU-20 groups (P<0.05). We also observed an increase in weight of abdominal fat in the diabetic mice compared to the control and probiotic groups.

**Table 3:** Effects of *B. toyo* SAU-20 on growth performance in HFD/STZ-induced T2DM mice.

Growth parameters	Groups		
	C	DBG	*Bacillus toyo*. SAU-20
Daily feed intake (g/day)	10.03 ± 0.80^b^	22.72 ± 2.21^a^	13.87 ± 0.80^b^
Daily water intake (ml/day)	7.49 ± 0.88^c^	14.61 ± 0.44^a^	11.35 ± 2.43^b^
Average body weight (g)	47.69 ± 0.79^a^	37.33 ± 2.02^c^	43. 96 ± 1.44^b^
Liver index (%)	2.24 ± 0.31^a^	1.42 ± 0.31^b^	2.19 ± 0.32^a^
Kidney index (%)	0.26 ± 0.03^a^	0.18 ± 0.04^b^	0.20 ± 0.05^b^
spleen index (%)	0.49 ± 0.03^a^	0.35 ± 0.04^b^	0.50 ± 0.05^a^
Abdominal fat index (%)	0.03 ± 0.01^c^	0.16 ± 0.05^a^	0.11 ± 0.03^b^

The table is represented as means value ± standard deviation (SD). values in the same row with different superscripts a-c are statistically different (n=8, P<0.05). C, normal group, blank control mice without HFD/STZ treatment; DBG, T2DM model mice with HFD/STZ treatment; DBG + B. toyo SAU-20, mice with HFD/STZ treatment and oral gavage administration of B. toyo SAU-20.

Organ indices, Organ weight/Body weight X 100%.

### Effects of *B. toyo* SAU-20 on Blood Glucose Increase and Oral Glucose Tolerance Test (OGTT) in HFD/STZ-Induced T2DM Mice

**Figure 1:**
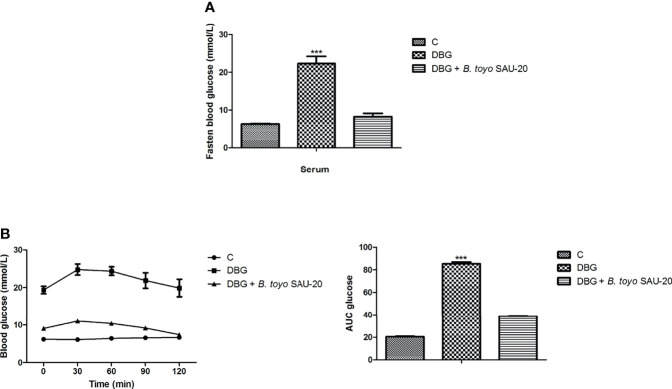
Effects of *B. toyo* SAU-20 on blood glucose increase in HFD/STZ-induced T2DM mice. **(A)** Fasting blood glucose (mmol/L) **(B)** Oral glucose tolerance test (mmol/L). Values were showed by mean ± Sd. ***P < 0.05 DBG compared with the Control (C) and DBG + *B. toyo* SAU-20 groups (n=6).

As showed in **Figure 1A**, the fasting blood glucose level of the DBG group after the 35-day administration period was higher as compared to the control (C) and *B. toyo* SAU-20 groups (P<0.05). However, there was no difference in fasting blood glucose between the control (C) and *B. toyo* SAU-20 groups. Furthermore, we observed a larger glucose area under the curve (AUC) in DBG mice as compared to the control (C) (**Figure 1B**, P<0.05). The glucose AUC was significantly lowered following oral administration of *B. toyo* SAU-20 to mice compared to the DBG (P < 0.05), with no significant difference compared to control (C) group (P>0.05).

### Effects of *B. toyo* SAU-20 on Biochemical Parameters in HFD/STZ Induced T2DM Mice

As shown in **Table 4**, the levels of AST, ALT, fructosamine, and HOMA-IR in the diabetic (DBG) group were significantly higher compared to those of the control (C) group whereas the insulin level was lower in the diabetic group (P<0.05). However, the oral administering of *B. toyo* SAU-20 significantly decreased the levels of biochemical parameters (AST, ALT, fructosamine, and HOMA-IR) and increase the levels of insulin compared to group DG mice (P<0.05). However, the frutosamine and HOMA-IR levels of the *B. toyo* SAU-20 group was significantly higher than the control (C) group.

**Table 4:** Effects of *B. toyonensis* SAU-20 on biochemical parameters in HFD/STZ-induced T2DM mice.

Tissue	Biochemical parameters	Groups		
		C	DBG	DBG + *B. toyo* SAU-20
Blood	AST (U/L)	130.67 ± 2.08^c^	253.0 ± 14.93^a^	201.33 ± 5.43^b^
	ALT (U/L)	28.67 ± 1.53^c^	114.67 ± 3.51^a^	65.33 ± 4.51^b^
	Fructosamine (μmol/L)	136.33 ± 3.51^b^	445.87 ± 92.62^a^	262.97 ± 7.42^b^
	Blood insulin (mU/L)	13.52 ± 0.39^a^	9.01 ± 0.73^b^	13.23 ± 2.12^a^
	HOMA-IR	3.80 ± 0.16^b^	8.91 ± 0.20^a^	4.82 ± 0.80^b^
Liver	Liver glycogen (mg/g protein)	4.80 ± 0.30^a^	1.57 ± 0.45^b^	4.10 ± 0.17^a^
**Lipid profiles**				
Blood	TC (mmol/L)	2.61 ± 0.56^c^	5.12 ± 0.21^a^	3.67 ± 0.21^b^
	TG (mmol/L)	1.40 ± 0.08^b^	2.74 ± 0.07^a^	1.56 ± 0.14^b^
	LDL-C (mmol/L)	2.41 ± 0.17^c^	4.37 ± 0.04^a^	3.07 ± 0.06^b^
	HDL-C (mmol/L)	0.23 ± 0.04^a^	0.10 ± 0.03^b^	0.20 ± 0.04^a^
Liver	TC (mmol/L)	2.13 ± 0.18^c^	4.71 ± 0.16^a^	3.07 ± 0.04^b^
	TG (mmol/L)	0.92 ± 0.07^c^	2.06 ± 0.07^a^	1.12 ± 0.06^b^
	LDL-C (mmol/L)	1.27 ± 0.06^b^	3.08 ± 0.06^a^	1.46 ± 0.11^b^
	HDL-C (mmol/L)	0.17 ± 0.02^a^	0.02 ± 0.01^b^	0.14 ± 0.03^a^
**Oxidative stress**				
Blood	MDA (nmol/mL)	0.54 ± 0.04^b^	1.82 ± 0.12^a^	0.67 ± 0.14^b^
	SOD (ng/mL)	9.45 ± 0.57^a^	5.69 ± 0.39^b^	9.10 ± 0.75^a^
	GSH (ng/mL)	1.42 ± 0.15^a^	0.49 ± 0.07^c^	0.90 ± 0.06^b^
Liver	MDA (nmol/mL)	0.59 ± 0.03^b^	0.88 ± 0.07^a^	0.60 ± 0.02^b^
	SOD (ng/mL)	7.55 ± 0.41^a^	4.18 ± 0.37^b^	7.83 ± 0.89^a^
	GSH (ng/mL)	1.05 ± 0.08^a^	0.33 ± 0.03^c^	0.84 ± 0.16^b^

Data represent mean ± SD (n = 5), values in the same row with different superscript letters are significantly different (P< 0.05). C, normal group, blank control mice without HFD/STZ treatment; DBG, T2DM model mice with HFD/STZ treatment; DBG + B. toyo SAU-20, mice with HFD/STZ treatment and oral gavage administration of B. toyonensis SAU-20.

AST, Aspartate Aminotransferase; ALT, Alanine Aminotransferase; TC, total cholesterol; TG, total triglyceride; LDL-C, LDL-cholesterol; HDL-C, HDL-cholesterol; GSH, glutathione; GSH-PX, glutathione peroxide; CAT, catalase; SOD, superoxide dismutase; MDA, malondialdehyde.

Liver glycogen content was lower in DBG group compared to the control (C) and *B. toyo* SAU-20 groups (P>0.05).

### Effects of *B. toyo* SAU-20 on Lipid Profile in HFD/STZ Induced T2DM Mice

The effects of *B. toyo* SAU-20 on the lipid profile are shown in **Table 4**. TC, TG and LDL-C levels in Diabetic group (DBG) were higher compared to control (C) group in both serum and liver (P< 0.05). However, these parameters were significantly attenuated in the *B. toyo* SAU-20 group (P< 0.05). Moreover, the TC (serum and liver), TG (Liver), LDL-C (serum) levels in the *B. toyo* SAU-20 group were increased as compared to the control (C) group (P<0.05). There were no significant differences in TG (serum) and LDL-C (liver) levels between *B. toyo* SAU-20 group and control (C) group. The HDL-C levels in the Diabetic group (DBG) were decreased as compared to the *B. toyo* SAU-20 group and control (C) group (P<0.05), however, there were no significant differences in HDL-C levels between *B. toyo* SAU-20 group and control (C) group.

### Effects of *B. toyo* SAU-20 on Antioxidant Parameters in HFD/STZ Induced T2DM Mice

The deviations in oxidative stress status of the mice livers are presented in **Table 4**. As compared to control (C) group, the oxidative stress component MDA increased in the DBG whereas the antioxidative stress components (SOD and GSH) in the DBG group was greatly decreased (P<0.05). However, the oral administration of *B. toyo* SAU*-*20 reverted these effects by increasing the levels of antioxidative stress components (SOD and GSH) and reducing the levels of oxidative stress component MDA (P<0.05). Moreover, the levels of antioxidative components (GSH) in the *B. toyo* SAU-20 group were significantly lower compared to the control group (P<0.05).

### Effects of *B. toyo* SAU-20 on Hepatic Steatosis in HFD/STZ Induced T2DM Mice

**Figure 2:**
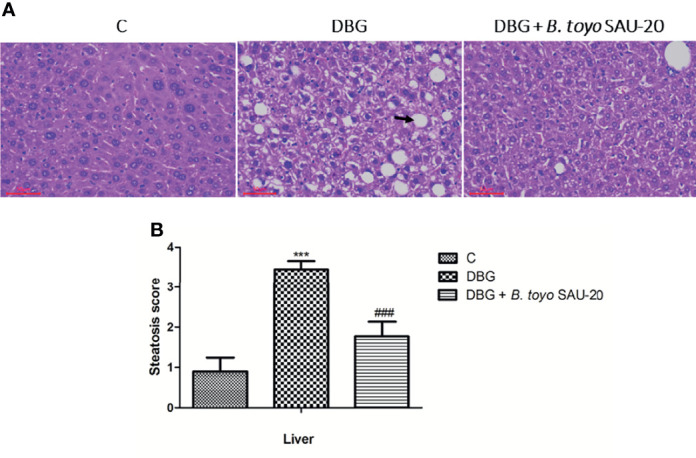
Effects of *B. toyo* SAU-20 on hepatic steatosis in HFD/STZ-induced T2DM mice. **(A)** shows the histological results of mice liver observed at magnification 200 ×. C- Liver section from control mice showed normal appearance of liver cells; DBG-Liver section from HFD.STZ mice showed marked fatty infiltration of hepatocytes with macro- or micro-vesicular steatosis (black arrow); DGB + *B. toyo* SAU-20- Liver section from *B. toyo* SAU-20 mice showed mild fatty infiltration of hepatocytes. The result shown here was from one representative experiment of four different samples with similar results (n=5). **(B)** Hepatic steatosis score. Values were showed by mean ± Sd. ***P < 0.05 DBG compared with the Control (C) and DBG + *B. toyo* SAU-20 groups. ^###^P < 0.05 DGB + *B. toyo* SAU-20 compared with the Control (C) group.

As represented in **Figure 2A**, we observed hepatomegaly, vesicular degeneration and prominent diffuse macrovesicular steatosis in livers of the diabetic mice (DBG) compared to the control group that showed normal histological appearance of the liver (C). However, oral administration of *B. toyo* SAU-20 reversed the accumulation of fats induced by HFD/STZ treatment (**Figure 2B**).

### Effects of *B. toyo* SAU-20 on Relative mRNA and Protein (ELISA) Expression of Genes Related Inflammation in Liver Tissues of HFD/STZ Induced T2DM Mice

**Figure 3:**
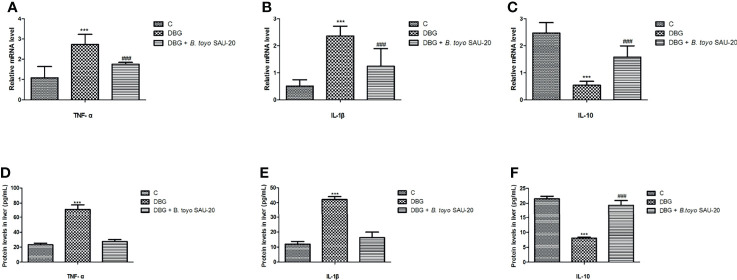
Effects of *B. toyo* SAU-20 on relative mRNA and protein (ELISA) expressions of pro- and anti-inflammation related cytokines in HFD/STZ-induced T2DM mice. **(A–C)** relative mRNA levels of pro- and anti-inflammatory cytokines **(D–F)** Protein levels of pro- and anti-inflammatory cytokines Values were showed as mean ± Sd. ***P < 0.05 DBG compared with the Control (C) and DBG + *B. toyo* SAU-20 groups. **^###^**P < 0.05 DGB + *B. toyo* SAU-20 compared with the Control (C) group (n=8).

As shown in **Figure 3**. The mRNA expression levels of genes of pro-inflammatory cytokines (IL-1β and TNF-α) were significantly elevated whereas anti-inflammatory cytokine (IL-10) were reduced in the diabetic (DBG) group compared to the control (**Figures 3A–C**, P<0.05). However, *B. toyo* SAU-20 reduced the expression levels of pro-inflammatory cytokines and increased the expression of anti-inflammatory cytokines compared to the DBG group (p<0.05) but the expression levels of both pro- and anti- inflammatory cytokines were different to the control (P<0.05). Similarly, the protein (ELISA) expression levels of pro-inflammatory cytokines were higher whereas the anti-inflammation cytokines were lower in the DBG group compared to the control and the *B. toyo* SAU-20 groups (**Figures 3D–F**). However, the protein level of IL-10 in the SAU-20 group was significantly lower that the control.

### Effects of *B. toyo* SAU-20 on Relative mRNA Expression of Genes Related to Lipogenesis and Fatty Acid Oxidation in Liver Tissues of HFD/STZ Induced T2DM Mice

**Figure 4:**
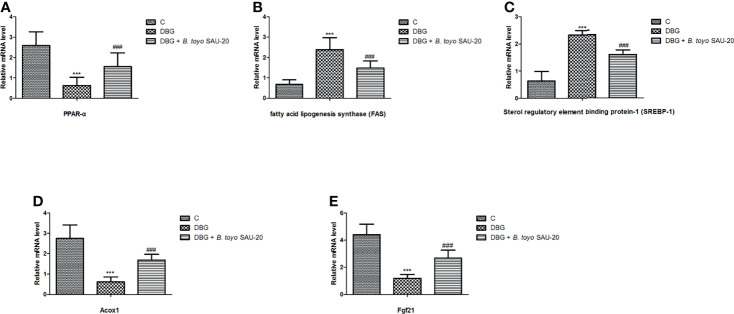
Effects of *B. toyo* SAU-20 on relative mRNA expressions of genes related to lipogenesis and fatty acid oxidation in liver tissues of HFD/STZ-induced T2DM mice. **(A–C)** Relative mRNA levels of genes responsible for lipogenesis. **(D, E)** Relative mRNA levels of genes responsible for fatty acid oxidation. Values were showed by mean ± Sd. ***P < 0.05 DBG compared with the Control (C) and DBG + *B. toyo* SAU-20 groups. **^###^**P < 0.05 DGB + *B. toyo* SAU-20 compared with the Control (C) group (n=8).

As shown in **Figure 4**. The mRNA expression levels of peroxisome proliferator activated receptor (PPARα) was decreased in the T2DM mice group compared to the control and the *B. toyo* SAU-20 group (**Figure 4A**, P<0.05). However, the levels of PPARα in the *B. toyo* SAU-20 group was significantly lower than the control group. In addition, the expression levels of genes related to lipogenesis, fatty acid lipogenesis synthase (FAS) and sterol regulatory element binding protein-1 (SREBP-1) were upregulated in the Diabetic (DBG) group compared to the control and the feeding of *B. toyo* SAU-20 downregulated these genes (**Figures 4B, C**, P<0.05), but the expression levels were higher compared to the control group (P<0.05). Furthermore, genes related to fatty acid oxidation, Acox1 and Fgf21 were downregulated in the DBG group compared to the control group but the oral administration of *B. toyo* SAU-20 reverted this effect by increasing the expression levels of Acox1 and Fgf21, however, was significantly lower than the control group (**Figure 4D**, P<0.05).

## Discussion

Numerous studies have reported on novel therapeutic agents for treating treatment of type 2 diabetes (19–23). One of the strategies that have been developed and in use is the oral administration of probiotics to modulate intestinal microbiota, which has been associated with the treatment or delay of the onset diabetes (24, 25). Lactobacillus and Bifidobacterium species have been reported to improve glucose tolerance, glucose-induced insulin secretion, stabilized inflammatory activities, and decrease the frequency of diabetes (26). In this study we tested the effect of *B. toyo* SAU-20 a novel probiotic bacteria isolated by our lab on a well-established diabetic mice model, which was induced by HFD feeding along with intraperitoneal STZ injection. The success of the T2DM mice model were measured by observing the usual phenotypes of T2DM such as weight loss, increased food and water consumption, hyperglycemia, and insulin resistance (27). Hepatic steatosis is a common disorder of T2DM (28, 29). Inequalities in hepatic lipid metabolism results in the accumulation of hepatic triglycerides and insulin resistance, which finally cause hepatic steatosis (30). Our results revealed that the body, liver, and spleen weights of the diabetic mice were lower than those of control group. These results were similar with previous reports by Zafar and Naeem-Ul-Hassan Naqvi, (31). Moreover, the administration of SAU-20 maintained the body weights and prevented weight loss by T2DM induced diabetes.

Impaired glucose tolerance is a key parameter for type 2 diabetes (32). Our results from fasten glucose and OGTT indicated that blood glucose levels in the diabetic group were elevated compared to the control. However, the oral administration of *B. toyo* SAU-20 reduced the glucose levels in the blood. These results demonstrated that oral administration of *B. toyo* SAU-20 may improve glucose tolerance and prevent the development of hyperglycemia in type 2 diabetic mice.

Various studies have reported that diabetes causes a rise in the AST and ALT levels (33, 34). Similarly, in this study we observed an increase in the levels of liver injury markers in the blood, however, the oral administration of *B. toyo* SAU-20 reduced the levels of these liver inflammation and damage markers. This result was similar to the study by Hsieh et al. (35) who reported that *Lactobacillus salivarius AP-32* and *Lactobacillus reuteri GL-104* (probiotic) could reduce the levels of AST and ALT in type 2 diabetes patients. Furthermore, we also observed that, the level of liver glycogen was much higher in the *B. toyo* SAU-20 group compared to the diabetic group, indicating that *B. toyo* SAU-20 could reduce blood glucose by excess glucose to glycogen in the liver. This result was similar with previous studies by Tao et al. (36).

Insulin is the major life-saving prescription for type 1 and type 2 Diabetes Mellites (37). Insulin plays active roles in growth performance, blood glucose and lipids maintenance and wound healing (38–41).

Insulin resistance was significantly higher whereas insulin levels were reduced in the diabetic mice compared to control group. However, *B. toyo* SAU-20 consumption increased the insulin levels and decreased the value of HOMA-IR index in HFD/STZ mice. These results indicated that *B. toyo* SAU-20 has the potential to increase production of insulin in HFD/STZ mice, thereby improving growth performance, insulin resistance, glucose tolerance and lipid profiles which was evident in our work.

The major pathogenesis of insulin resistance is the buildup of visceral fat which promotes increase blood pressure, dyslipidemia, and dysregulation of glucose metabolism (42). Amongst the target tissues of insulin, liver is the principal regulator of lipid metabolism *via* regulating lipogenesis (43). From our study, we observed the oral administration of *B. toyo* SAU-20 decreased the levels key constituents of metabolic syndrome, including serum glucose, insulin resistance, LDL, TG, and cholesterol, enhanced by HFD/STZ in both the serum and liver. Therefore, we concluded that oral administration of *B. toyo* SAU-20 could improve hepatic steatosis by reducing the production lipids in both the serum and hepatic tissues.

Oxidative stress is associated with hepatic steatosis and T2DM (44). Hyperglycemia and dyslipidemia could increase the production of reactive oxygen species (ROS) which may cause damage to living cells and specific receptors of the cell membrane and then finally cause injury to the organs such as islets of Langerhans and liver (45, 46). Several probiotics have been reported to show effective antioxidants activities (47). In this study, feeding of *B. toyo* SAU-20 significantly increased the antioxidant activities and decreased oxidative stress activity (MDA) in TD2M mice. These results indicated that probiotic strain *B. toyo* SAU-20 can reduce the activity of ROS, thus improving hepatic steatosis, insulin resistance, and preventing the damage to organs such as the pancreas, the liver, and the kidney.

Systemic and subclinical inflammation has been reported to play a vital role in Type 2 diabetes mellitus (48, 49). Our results showed that the expressions of pro inflammatory cytokines (IL-1β and TNF-α) in the liver was elevated while the level of anti-inflammatory (IL-10) was reduced in the diabetic group compared to the control group, however, the oral administration of *B. toyo* SAU-20 reverted these effects. This result was in consistent with the study by Liu et al. (50) who showed that *Lactobacillus rhamnosus* GG culture supernatant (LGGs) could reduce liver inflammation and injury in High-fat/high-fructose diet plus intermittent hypoxia exposure-induced metabolic dysfunction.

To reveal the possible mechanisms of *B. toyo* SAU-20 in suppression of hepatic lipid accumulation in TD2M mice, we evaluated the liver mRNA levels of genes involved in lipogenesis. Srebp-1c is a well-known transcription factor that regulates hepatic fatty acid and triglyceride biosynthesis by upregulating the expression of key genes, such as FAS (51). Fatty acid synthase (FAS) is a multifunctional enzyme involved in the production of fatty acids for lipid biosynthesis and is overexpressed in multiple diseases like cancer, viral, nonalcoholic fatty liver disease, and metabolic disorders (52). HFD/STZ treatment has been reported to elevates the expression of hepatic Srebp-1c, and FAS in rats (53). Our study showed that HFD/STZ treatment remarkably induced the expression of lipogenic genes (srebp-1c and FAS) in mice liver. However, the oral administration *B. toyo* SAU-20 counteracted the increase in hepatic lipogenic genes in HFD/STZ mice. This result was similar to the work of Hsieh et al. (3) who reported that *Lactobacillus reuteri* GMNL-263 could downregulate genes related to lipogenesis in liver of rats fed high fructose diet. These results suggested that *B. toyo* SAU-20 consumption suppressed hepatic lipid accumulation in TD2M mice.

Hepatic fatty acid oxidation is associated with fatty acid catabolism along with diabetic pathogenesis (54, 55). The peroxisome proliferator-activated receptors (PPARs) control the expression of key genes related to metabolic diseases including obesity, dyslipidemia and diabetes (56). PPAR-α is mainly expressed in the liver and is associated with maintaining lipid homeostasis (57). It promotes fatty acid β-oxidation by modulating the respective genes (58) as well as improve glucose metabolism in T2DM by increasing insulin sensitivity and reducing hyperglycemia and hyperinsulinemia (59). TG and low-density lipoprotein metabolism is reduced by PPAR-α activation (60). while the knockout reduce the expression of genes involved in fatty acid oxidation in the liver (6). Acox1 and Fgf21 are genes responsible for encoding the fatty acid oxidation enzymes and are regulated by PPAR-α (6). We found that *B. toyo* SAU-20 upregulated the expression of PPAR-α and its target genes involved in fatty acid oxidation (Acox1 and Fgf21) in the liver of diabetic mice. Therefore, one alternative therapeutic approach for diabetes could be the amelioration of hepatic steatosis by activating PPAR-α pathway. However, this requires further research.

In conclusion, this study demonstrates that *B. toyo* SAU-20 improved lipid profiles and attenuated hepatic steatosis in HFD/STZ-induced diabetic mice. The possible mechanism for these effects might be associated to *B. toyo* SAU-20 ability to decrease insulin resistance and oxidative stress, improve lipid profiles, downregulate genes responsible for lipogenesis, and upregulating genes responsible for fatty acid oxidation. These results support the use of *B. toyo* SAU-20 for the primary treatment of T2DM and other fat related metabolic disorders, however, there still the need for further studies to validate its use through various clinical trials.

## Data Availability Statement

The original contributions presented in the study are included in the article/supplementary material. Further inquiries can be directed to the corresponding author.

## Ethics Statement

The animal study was reviewed and approved by Institutional Animal Care and Use Committee of Sichuan Agricultural University, Sichuan, China, under the permit number DKY-B2019603005.

## Author Contributions

ZR, SO, LX, JWe, ZC, and JWa: Conceptualization, Methodology, Software. ZR, SO, LX, JWe, JWa, ZC, and XN: Data collection, Writing, Original draft preparation. SO, JWe, ZC, and JD: Validation, Investigation. ZR, SO, JWe, XN, JD and YH: Review editing, Funding, Supervision. All authors have read and agreed to the published version of the manuscript.

## Funding

This research was supported by Sichuan Province Science and Technology Support Program (Grant No. 2020YFS0337).

## Conflict of Interest

YH is/was employed by New Ruipeng Pet Healthcare Group Co., Ltd., Shenzhen 518000, China.

The remaining authors declare that the research was conducted in the absence of any commercial or financial relationships that could be construed as a potential conflict of interest.

## Publisher’s Note

All claims expressed in this article are solely those of the authors and do not necessarily represent those of their affiliated organizations, or those of the publisher, the editors and the reviewers. Any product that may be evaluated in this article, or claim that may be made by its manufacturer, is not guaranteed or endorsed by the publisher.
